# Cost-effective core@shell structured zero-valent iron nanoparticles @ magnetic (nZVI@Fe_3_O_4_) for Cr(vi) removal from aqueous solutions: preparation by disproportionation of Fe(ii)†

**DOI:** 10.1039/d3ra03133k

**Published:** 2023-09-08

**Authors:** Chuan He, Yarong Ding, Canhua Li, Wang Yan, Aiqin Mao, Shuxian Wei, Minghui Li

**Affiliations:** a College of Metallurgical Engineering, Anhui University of Technology Ma'anshan 243000 China licanhua1979@163.com; b Jiuquan Vocational and Technical College Jiuquan 735000 China; c School of Materials Science and Engineering, Anhui University of Technology Ma'anshan 243000 China; d Xuancheng Industrial Technology Research Institute, Anhui University of Technology Xuancheng 242002 China

## Abstract

Nanoscale zero-valent iron (nZVI) and its composites are known for their excellent ability to remove Cr(vi), but their preparation can be expensive due to the reduction processes. This study presents a cost-effective method to prepare core@shell structured nZVI@Fe_3_O_4_ nanocomposites using a novel Fe(ii) disproportionation reaction. The nZVI@Fe_3_O_4_ was thoroughly characterized using various techniques, including FESEM, HRTEM, EDS, XPS, XRD, FTIR, and VSM. Batch experiments were performed to evaluate the removal efficiency of nZVI@Fe_3_O_4_ in eliminating Cr(vi) ions from aqueous solutions, while classical models were employed to investigate the influencing factors associated with the removal process. The results showed that a 0.7 mg per ml NaOH solution reacted with Fe(ii) at 150 °C for 0.5 h could be used to prepare nZVI@Fe_3_O_4_ composites efficiently and inexpensively. nZVI@Fe_3_O_4_ was able to remove more than 99% of Cr(vi) from both simulated Cr(vi) solutions and real electroplating wastewater, and the recovery and preparation could be easily performed using external magnets to separate it from the solution. At pH 6.0, the maximum adsorption capacity (*q*_max_) for Cr(vi) reached 58.67 mg g^−1^. The reaction mechanism was discussed from the perspective of electron transfer. Overall, the results suggest that nZVI@Fe_3_O_4_, an efficient adsorbent prepared using an environmentally friendly and inexpensive Fe(ii) disproportionation reaction, is a promising option for the treatment of Cr(vi) from industrial wastewater and other contaminated water sources.

## Abstract 2

Nanoscale zero-valent iron (nZVI) and its composites are known for their excellent ability to remove Cr(vi), but their preparation can be expensive due to the reduction processes.

## Graphical abstract

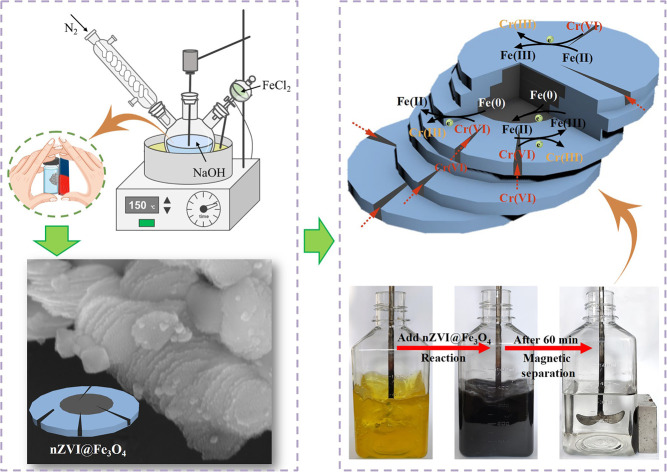

## 1. Introduction

Pollution is a major global environmental issue that leads to illness and premature death.^1^ One of the most dangerous contaminants found in polluted sites is chromium, which frequently appears in both trivalent (Cr(iii)) and hexavalent (Cr(vi)) forms.^2^ While Cr(vi) is primarily caused by human activities, such as industrial manufacturing and metallurgy, Cr(iii) is often insoluble in aqueous solutions and soil.^3^ The soluble forms of Cr(vi), which have a high mobility in nature, are particularly harmful to human health, causing respiratory and eye irritation, dermatitis, gastrointestinal damage, and even cancers like lung and kidney cancer.^4,5^

Adsorption is recognized as an effective and cost-effective approach for treating heavy metals such as Cr(vi), and it involves using substances like low-valent iron (Fe(0) and/or Fe(ii)) to act as a trapping and reducing agent.^6^ These substances have been shown to excel at capturing Cr(vi) in groundwater, industrial wastewater, and soil. Once captured, Cr(vi) is converted to Cr(iii) and rendered immobile by reacting with Fe to form insoluble compounds, which is environmentally safe.^7,8^ Nano-magnetite (nFe_3_O_4_) and nano zero-valent iron (nZVI) are two common substances that possess magnetic properties and are frequently utilized for adsorption purposes. The magnetic properties of these substances are a critical factor in their selection as nFe_3_O_4_ and nZVI can be easily separated by magnetic separation, which is often more economical and convenient compared to complex membrane filtration.^9^ Among these substances, nZVI has a higher reduction potential and is widely used for soil and groundwater remediation in Europe and the United States.^10^ However, there are two significant challenges in using nZVI for adsorption. Firstly, acquiring nZVI involves costly and complex “top-down” or “bottom-up” processes that may lead to secondary contamination.^11,12^ Secondly, nZVI particles tend to rapidly agglomerate into larger particles due to interparticle interactions, and surface passivation due to high oxidation environments can result in a decrease in reactivity.^13^ These challenges can reduce the effectiveness of nZVI in adsorption, and researchers are working to address these issues to improve the use of nZVI for heavy metal treatment.

Many methods have been developed to modify and stabilize nZVI in order to maximize its activity and utilization. These methods include carbon-supported,^13^ phosphorylation,^14^ sulfidation,^15^ bimetallization,^16^ encapsulation.^17^ In recent years, the formation of composites with nFe_3_O_4_ has received significant attention due to the capability of nZVI to transfer electrons to the surface of Fe_3_O_4_, which can regenerate Fe(iii) to Fe(ii) and improve electron transfer.^18–23^ However, most of these methods involve a two-step process starting with the preparation of nFe_3_O_4_ using co-precipitation, nZVI is typically ready by reducing Fe(ii) to Fe(0) using NaBH_4_ or high-temperature reduction with C and H_2_.^24,25^ Some studies have used the disproportionation reaction of Fe(ii) to prepare Fe@Fe_3_O_4_ composites, but these typically involve complicated precursors like calcite (FeO) and have not been widely used for contaminant removal applications.^26,27^

In this study, we used a very inexpensive and simple procedure to get rid of the expensive and complicated reduction process. nZVI@Fe_3_O_4_ was synthesized through a one-step process, using ferrous chloride precursors and allowing Fe(ii) disproportionation to occur in hot concentrated lye. The NaOH concentration and reaction time of the process were investigated. The fine features and physical/chemical properties of the products were studied by performing FESEM, HRTEM, EDS, XRD, XPS, BET, VSM, FTIR characterization. Following that, the removal efficiency was evaluated under varying initial pH levels. Kinetics, thermodynamics, and adsorption isotherms were analyzed by studying the effect of different initial Cr(vi) concentrations, dosing levels, and temperature on removal efficiency. These analyses helped to elucidate the removal process, the capacity of removal, and the spontaneity of the reaction. Furthermore, the capability of nZVI@Fe_3_O_4_ to treat real electroplating wastewater was examined. Finally, based on the identification of the reaction products and the assessment of the adsorption behavior, possible reaction mechanisms were further suggested. The outcomes of this study could potentially broaden the range of applications for nZVI and Fe_3_O_4_ composites, particularly in the treatment of Cr(vi)-containing wastewater.

## 2. Materials and methods

### 2.1. Materials

Ferrous chloride tetrahydrate (FeCl_2_·4H_2_O), potassium dichromate (K_2_Cr_2_O_7_), sodium hydroxide (NaOH), and all the other chemical agent were purchased from the Sinopharm Group Chemical Reagent Co., Ltd, China. In all experiments, deionized water was utilized, and the water used to prepare the adsorbent was deoxygenated by charging it with N_2_ gas for 0.5 h. The real plating wastewater is taken from a chrome metal plating line in the Fanchang Plating Center in Wuhu, China.

### 2.2. Preparation of nZVI@Fe_3_O_4_

The synthesis of the material was performed in a multi-necked flask which was in an oil bath, the temperature was programmed to 150 °C and the oxygen-free environment was strictly controlled. Briefly, 200 ml of NaOH solutions with various concentrations (0.5, 0.55, 0.6, 0.65, 0.7, 0.75 g ml^−1^) were made, and when the temperature of the NaOH solution increased to 145 °C, the prepared FeCl_2_ solution (In 20 ml of deionized water, FeCl_2_·4H_2_O dissolved with a mass of 9.941 g) was added dropwise to the high temperature NaOH solution, the reaction times were set to 0.5, 1.5, and 3 h, respectively, and a gradual change of the solution from grey to black could be observed. Once the reaction was completed, the products were promptly separated using magnets and rinsed multiple times with oxygen-free water, dried under vacuum and then used.

### 2.3. Analysis methods

The detail of analytical methods are shown in ESI.†

### 2.4. Adsorbent characterization

To prepare a stock solution of Cr(vi), 2.829 g of K_2_Cr_2_O_7_ was dissolved in water and then diluted to 1 L, resulting in a 1 g L^−1^ solution of Cr(vi), and the simulated wastewater with varying concentrations of Cr(vi) was prepared by diluting the stock solution. Batch experiments were conducted in open flasks at 25 °C by dispensing a certain concentration of Cr(vi) (500 ml per flask), adjusting the pH range using 0.1 M HCl or NaOH, and then adding a predetermined amount of nZVI@Fe_3_O_4_ to each flask, which was mixed immediately by shaker. At regular intervals, 5 mL aliquots of the solution were extracted and the particles were filtered out using a 0.45 μm membrane for measuring the solution concentration. To ensure effectiveness and accuracy of the results, all experiments were performed in triplicate, with consistently reproducible outcomes. The reported data represents the average values of three replicated experiments, with error bars indicating the standard deviation of the averages. The removal efficiency (RE%) of Cr(vi) was calculated using the following eqn (1):1
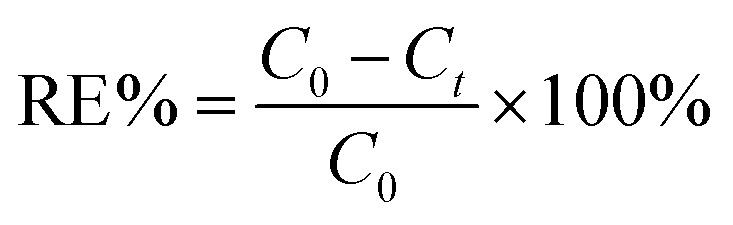
where *C*_0_ is the initial concentration (mg L^−1^) of Cr(vi), and *C*_*t*_ is the concentration (mg L^−1^) at a specific time. Meanwhile, the Cr(vi) removal capacity of nZVI@Fe_3_O_4_ is expressed in terms of the adsorption amount qt, which is calculated using eqn (2):2
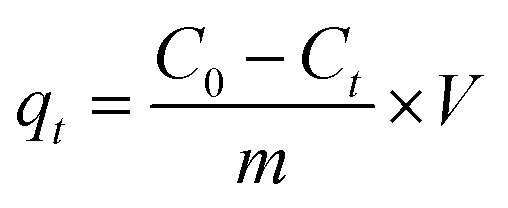
where *m* (g) is the nZVI@Fe_3_O_4_ dosage, and *V* (L) is the volume of Cr(vi) solution.

## 3. Results and discussion

### 3.1. Material synthesis

**Fig. 1:**
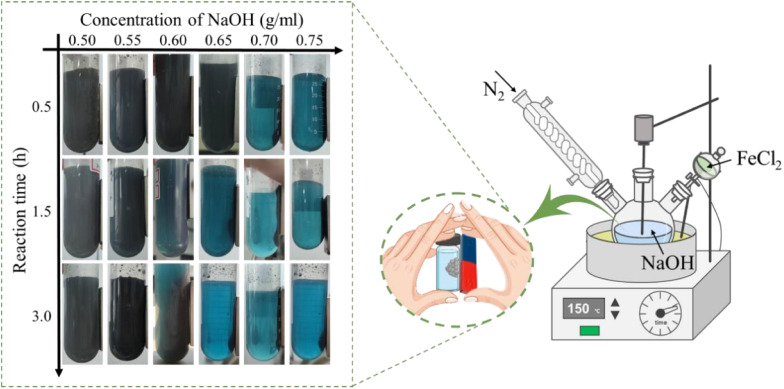
Preparation of nZVI@Fe_3_O_4_ by disproportionation of Fe(ii) using high concentration of NaOH solution, right: schematic diagram of the device, left: the extent of the reaction at each time for different NaOH concentrations was recorded by placing a magnet on one side of the test tube for 10 seconds and taking pictures.

We investigated the impact of varying NaOH solution concentrations and reaction times on the formation of this material, and the state of the product could be judged using a magnet, as shown in Fig. 1. It was observed that a small amount of black magnetic material was produced when the NaOH concentration reached 0.5 mg ml^−1^. After further increasing the concentration to 0.6 mg ml^−1^ and prolonging the reaction time to 3 h, it was observed that most of the precipitation could be attracted to one side of the sample flask by the magnet, more magnetic phases were generated in the reaction system. In order to reduce energy and raw material consumption, subsequent characterization and experiments were conducted using a 0.7 mg per ml NaOH solution with 0.5 h reaction, which was sufficient for complete disproportionation of all the precipitates.

Before determining the concentration of NaOH in the Fig. 1, we conducted experiments with low concentrations of NaOH, but unfortunately, no disproportionation reaction occurred and the product was a gray-green ferrous hydroxide precipitate. However, under the conditions of high concentration of OH^−^, the boiling point of the solution will be increased (the temperature of the reaction system was measured to be 147 °C), and when Fe(ii) is added dropwise, Fe(OH)_2_ is produced under oxygen-free conditions (eqn (3)), but Fe(OH)_2_ itself is unstable and is converted to FeO under the high temperature of a strong base (eqn (4)). At low temperatures (compared to higher temperatures), FeO is an unstable phase and spontaneously disproportionates, and then Fe_3_O_4_ and iron monomers are produced in a 1 : 1 molar ratio (eqn (5)).^28^

3 Fe^2+^ + 2OH^−^ → Fe(OH)_2_

4 Fe(OH)_2_ → FeO + H_2_O

5 4FeO → Fe + Fe_3_O_4_

### 3.2. Characterization of nZVI@Fe_3_O_4_

#### 3.2.1. XRD and XPS analysis

**Fig. 2:**
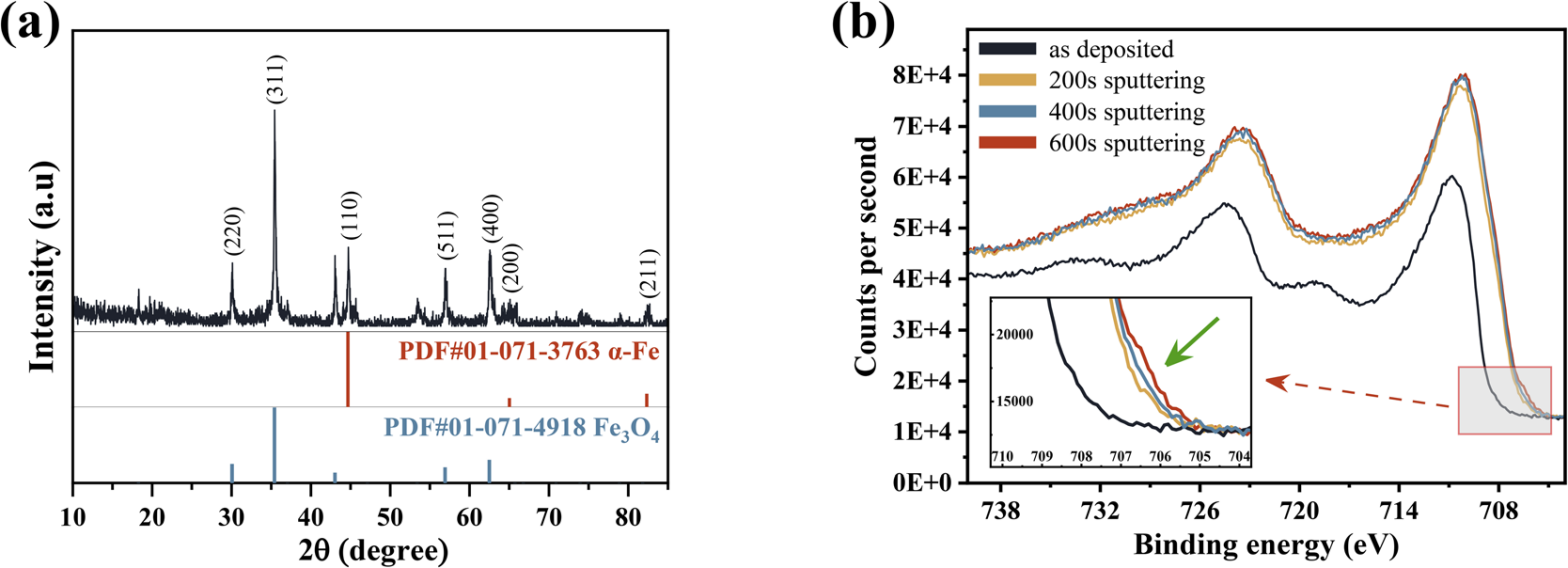
(a) XRD pattern and (b) Fe 2p high-resolution XPS spectra of nZVI@Fe_3_O_4_ before and after Ar^+^ sputtering.

Fig. 2 shows the XRD and XPS spectra of nZVI@Fe_3_O_4_ nanocomposites. In Fig. 2(a), the diffraction peaks representing the (110), (200), and (211) planes of α-Fe are marked in red at 44.67°, 65.02°, and 82.33°, respectively, as per the PDF#01-071-3763.^25^ While the peaks at 2*θ* = 30.07°, 35.4° and 62.5° associate to the characteristic diffraction peaks of Fe_3_O_4_ (PDF: 01-071-4918).^22^ This suggests that the nanoparticles prepared by Fe(ii) disproportionation reaction are mainly composed of α-Fe and Fe_3_O_4_, and it does not exclude that some Fe_3_O_4_ is derived from the oxidation of metallic iron.

The average crystalline size of α-Fe microcrystals calculated using Scherrer's formula described in the ESI† is 49 nm, which is similar to the already reported size of α-Fe microcrystals prepared by FeO disproportionation decomposition (53 nm).^27^

Meanwhile, the calculated average size of Fe_3_O_4_ microcrystals is 40 nm. It is worth mentioning that the sharp and narrow XRD diffraction peaks of α-Fe confirms the good crystallinity, unlike the nZVI prepared by liquid-phase reduction, whose cores often contain iron with poor crystallinity.^18^ As per prior studies, good crystallinity can enable the formation of effective electronic networks, which have the potential to improve conductive behavior when dealing with heavy metal ions and enhance material reactivity upon adsorption.^29,30^

XPS analysis gives a more detailed chemical characterization of the as-synthesized sample. Fig. 2(b) shows that the spin–orbit splitting Fe 2p peaks are observed at 706.8 and 723.5 eV In the pristine nZVI@Fe_3_O_4_, and these signals consist of photoelectrons from Fe(iii), Fe(ii) and Fe(0), respectively.^31^ The presence of Fe(0) is responsible for the peak observed at 706.8 eV, and after Ar^+^ sputtering, an increase in the signal of Fe(0) can be observed (green arrow inset in Fig. 2(b)), but the increase is very weak, indicating that α-Fe is not dispersed and precipitated in the matrix of Fe_3_O_4_, which is consistent with the result of nZVI@Fe_3_O_4_ nanocomposites prepared by FeO decomposition.^27^ Therefore, the nZVI@Fe_3_O_4_ may have a core@shell structure, where Fe(0) is located at the core position, resulting in the failure of the Fe(0) photoelectron to escape. The HRTEM and the slower removal rates of the subsequent batch tests likewise confirm this.

#### 3.2.2. FESEM-EDS and HRTEM analysis

**Fig. 3:**
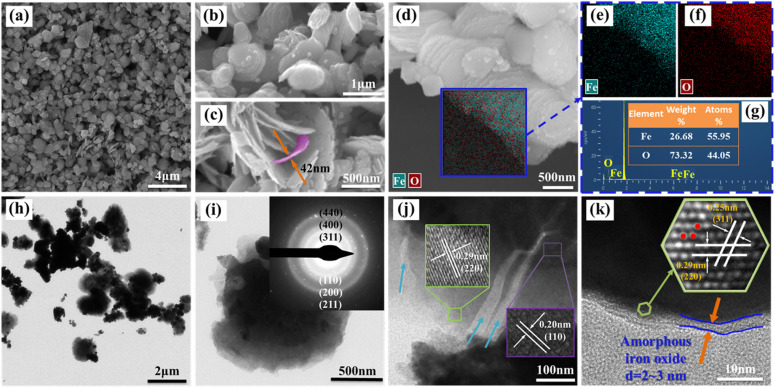
(a–d) FESEM images with different magnifications, (e and f) EDS mapping, (g) EDS elemental spectra with (d) surveys, (h and i) TEM images, and the inset shows SAED pattern, (j and k) HRTEM images of nZVI@Fe_3_O_4_.

The morphological characteristics and microstructural/chemical homogeneity of the as-synthesized particles were observed by FESEM/EDS and TEM/HETEM, as shown in Fig. 3. Fig. 3(a) and (b) display particles with a size distribution that is relatively homogeneous, with diameters ranging from 500 to 1500 nm. Furthermore, these particles consist of nanosheets, each being less than 50 nm thick, due to their surface energy and magnetic interactions, resulting in several or dozens being stacked on top of each other, as shown in the high magnification FESEM images in Fig. 3(c) and (d).^32^ This is consistent with the previous report on the thermal decomposition of galena to produce layered structure α-Fe/Fe_3_O_4_ composite particles.^33^ Moreover, the EDS shows that the main elements of the as-synthesized particles are Fe and O, the highest peak in Fig. 3(g) is contributed by the single-crystal silicon substrate, and no other impurities were found. The molar ratio of Fe : O is 55.95 : 44.05. In addition, the EDS elemental mappings shown in Fig. 3(e) and (f) demonstrate a uniform distribution of Fe and O elements across the surface of the particles.

The TEM/HRTEM were used to characterize the fine features of the nanosheets contained in the as-synthesized particle. Fig. 3(h) and (i) displays the a TEM images of the nanosheets, and the nanosheets have a darker central part (core) and a lighter outer part (shell), which is also confirm the core–shell structure of as-synthesized nZVI@Fe_3_O_4_ nanocomposites. Furthermore, the SAED pattern as inset in Fig. 3(i) shows several diffraction rings from inside to outside, which index to the crystallographic diffraction of (311), (400), and (440) of Fe_3_O_4_, as well as α-Fe (110), (200), and (211).^34^ In addition, the HRTEM image in Fig. 3(j) reveals a high degree of crystallinity of as-synthesized nanosheet, and the core has a calculated lattice spacing of 0.20 nm corresponding to the (110) plane of body-centered cubic α-Fe, while the shell has a observed lattice spacing of 0.29 nm, consistent with the (220) crystal plane of Fe_3_O_4_. Fe_3_O_4_ possesses an inverse spinel structure that can host Fe(ii) and Fe(iii) in octahedral sites and enables efficient electron transfer between them. As a result, Fe_3_O_4_ is a highly conductive semiconductor and is expected to function as a catalytic surface when deposited onto Fe(0).^30^ And the large number of fissures and defects between the nanosheets are beneficial for adsorbing heavy metal ions. Moreover, as seen in Fig. 3(k), the edges of the nanosheet display translucent amorphous nano-sized structures with thicknesses between 2 and 3 nm. The extremely thin size of Fe_3_O_4_ layers with disordered and defective nature also provide effective adsorbing sites for heavy metal ions.

#### 3.2.3. BET and VSM analysis

**Fig. 4:**
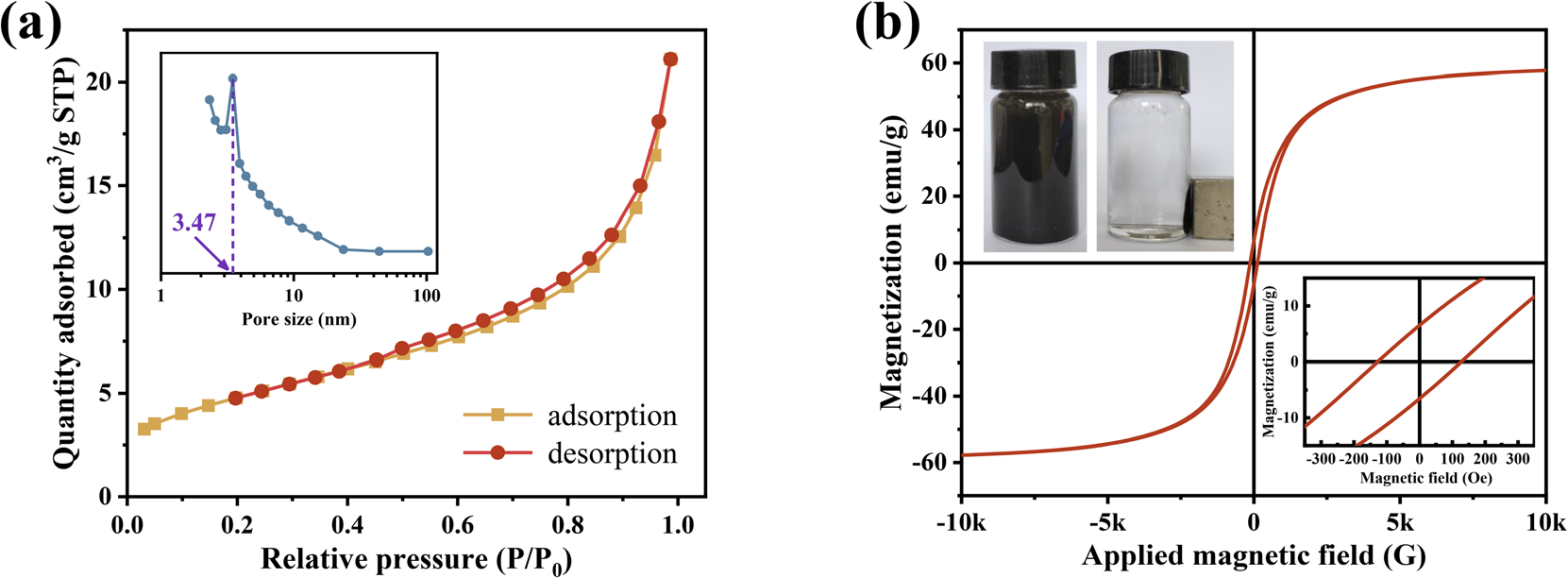
(a) N_2_ adsorption–desorption isotherms and pore size distribution diagram of nZVI@Fe_3_O_4_; (b) hysteresis loops of nZVI@Fe_3_O_4_ nanocomposite (top left: separation using magnets; bottom right: enlargement of the central region of the loop line).

The N_2_ adsorption–desorption behaviour of the nZVI@Fe_3_O_4_ nanocomposites is illustrated in Fig. 4(a). The isotherms exhibit closed curves with short platforms, inflection points, and hysteresis loops, which is the characteristic of a combination of type II and IV isotherms.^35,36^ The adsorption curves display an increase in adsorption at lower relative pressures, with a slight upward curve, consistent with a type II isotherm. This type of isotherm is typical of physical adsorption processes on nonporous adsorbents. The nZVI@Fe_3_O_4_ has a calculated specific surface area of 17.13 m^2^ g^−1^. Moreover, the pore size distribution profile exhibits a relatively sharp distribution of mesopores around 3.47 nm. The isotherms display an adsorption hysteresis similar to type IV isotherms at relative pressures above 0.4, and the hysteresis loop is ascertained as type H3-type according to the IUPAC classification, aggregates of plate-like particles often exhibit this type of isotherm caused by capillary condensation phenomena in the slits between them.^37^

The room-temperature magnetic hysteresis loops obtained from VSM measurements at a maximum magnetic field of 10 kOe are shown in Fig. 4(b). The magnetization curve exhibits a typical hysteresis loop at room temperature, demonstrating the ferromagnetic behavior of as-synthesized nZVI@Fe_3_O_4_ adsorbent nanocomposites. The magnetization curve of nZVI@Fe_3_O_4_ is very smooth, which suggests that the two magnetic phases (α-Fe and Fe_3_O_4_) are exchange-coupled.^27^ This is in contrast to the presence of a shoulder on the magnetization curves of multiple magnetic phases that lack exchange coupling. The saturation magnetization (*M*_S_) of the nZVI@Fe_3_O_4_ nanocomposites is 49.84 emu g^−1^, which is greater than that of nZVI coated with nonmagnetic materials.^38^ Additionally, nZVI@Fe_3_O_4_ has a coercivity (*H*_C_) of 127.28 Oe and a residual magnetization strength (*M*_r_) of 6.65 emu g^−1^. This magnetic characteristic of the as-synthesized nZVI@Fe_3_O_4_ nanocomposites enables it to easily separate from aqueous solution using the external magnetic field.

### 3.3. Study on the adsorption of Cr(vi) by nZVI@Fe_3_O_4_

#### 3.3.1. Effect of initial pH

**Fig. 5:**
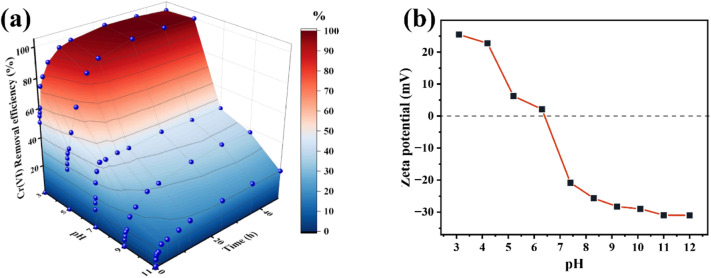
(a) pH-dependent Cr(vi) removal efficiency over the nZVI@Fe_3_O_4_ (initial Cr(vi) concentration 50 mg L^−1^, nZVI@Fe_3_O_4_ dosage 1 g L^−1^, 298.15 K); (b) zeta potential of nZVI@Fe_3_O_4_ at different pH.

The adsorption behaviour of Cr(vi) by nZVI@Fe_3_O_4_ was strongly influenced by the initial pH of the solution, as demonstrated in Fig. 5(a). The removal efficiency of Cr(vi) by nZVI@Fe_3_O_4_ was found to increase as the pH of the solution decreased from 11.0 to 3.0. At an initial pH of no more than 5.0, complete adsorption of Cr(vi) by nZVI@Fe_3_O_4_ was observed. However, as the pH increased, the efficiency of Cr(vi) removal decreased gradually, resulting in 37.25% (pH = 9.0) and 20.16% (pH = 11.0) respectively.

Explain this phenomenon by the surface chemistry of the aqueous phase, At low pH (1.0–6.5), the main species of Cr(vi) is HCrO_4_^−^, while the pH = 6.5–7.5, the species changes to CrO_4_^2−^ and Cr_2_O_7_^2−^, and pH > 7.5, CrO_4_^2−^ is the only existing form.^18^ Compared with CrO_4_^2−^ or Cr_2_O_7_^2−^, HCrO_4_^−^ is advantageous for electrostatic adsorption due to its lower free energy of adsorption at low pH.^39^ In another light, the surface charge of nZVI@Fe_3_O_4_ is determined by the pH. The surface of metal oxides typically contains hydroxyl groups that exhibit different forms depending on the pH of the solution. Fig. 5(b) shows that the zero potential point (pH_zpc_) of nZVI@Fe_3_O_4_ is about 6.5. When the pH is below pH_zpc_, the surface of nZVI@Fe_3_O_4_ is positively charged, making it favorable for adsorption of Cr(vi) anions.

#### 3.3.2. Adsorption kinetics

**Fig. 6:**
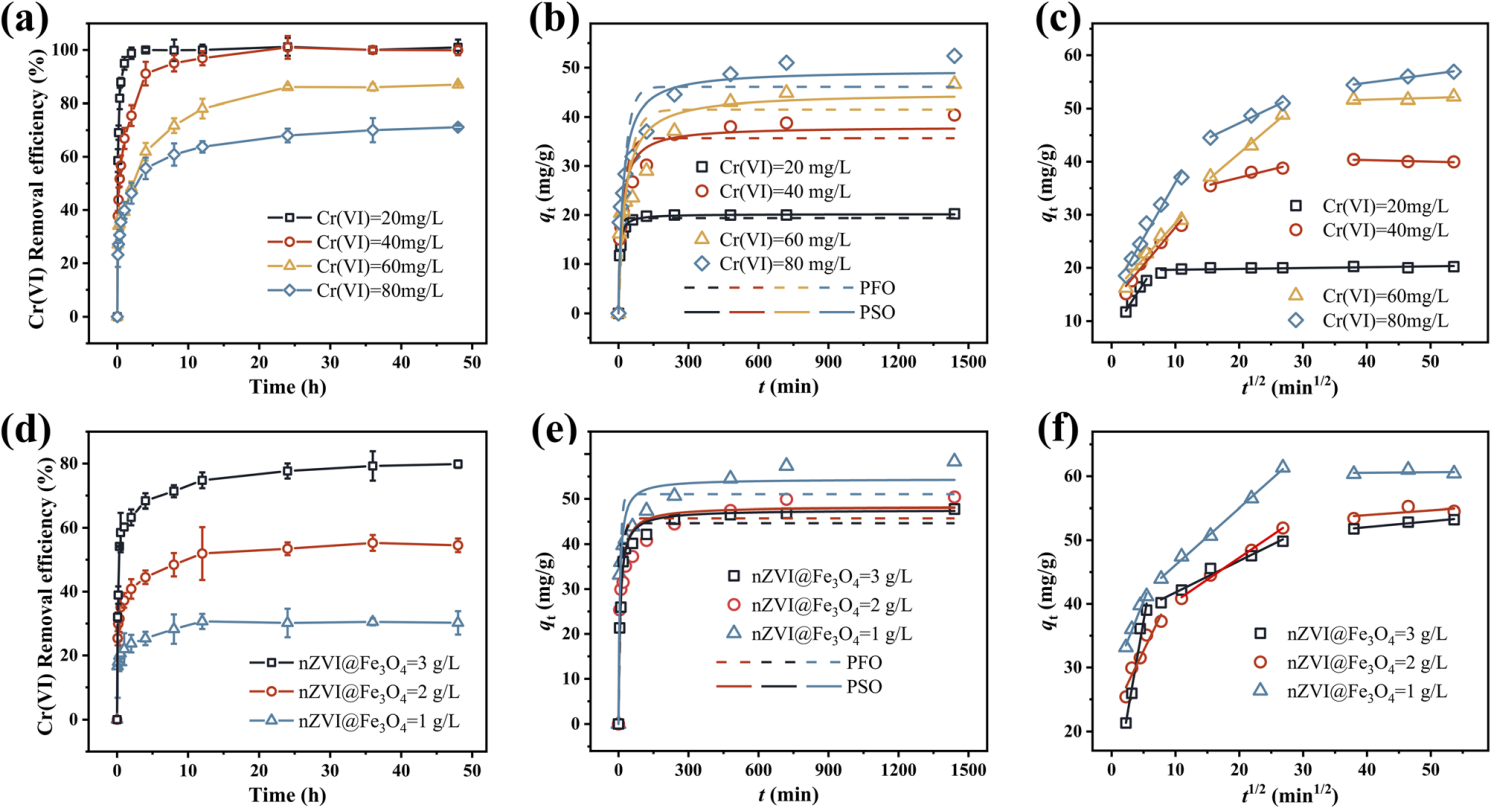
(a)–(c) Removal efficiency, PFO/PSO kinetics and internal diffusion model fits for different initial concentrations of Cr(vi), respectively; (d)–(f) removal efficiency, PFO/PSO kinetics and internal diffusion model fits for different nZVI@Fe_3_O_4_ dosing amounts, respectively.

Experiments were carried out to investigate the influence of both initial Cr(vi) concentration, ranging from 20 to 80 mg L^−1^, and nZVI@Fe_3_O_4_ dosage, ranging from 1 to 3 g L^−1^, on the removal efficiency of Cr(vi), as illustrated in Fig. 6(a) and (d), afterward, the adsorption kinetics of the removal process were analyzed (Fig. 6(b), (c), (e) and (f)). The maximum slope of the Cr(vi) removal efficiency curve was observed within the first 5 min after nZVI@Fe_3_O_4_ addition, which could be attributed to the initial adsorption in the reaction system, resulting from the large number of available adsorption sites on nZVI@Fe_3_O_4_. At a Cr(vi) concentration of 20 mg L^−1^, nZVI@Fe_3_O_4_ could completely remove the contaminant within a mere 2 h at a dosage of 1 g, rendering it undetectable by the measuring instrument. Even at a higher concentration of 40 mg L^−1^, the removal efficiency of the nZVI@Fe_3_O_4_ remained close to 100%, although the required time for complete removal was longer (Fig. 6(a)). When exposed to a concentration of 200 mg per L Cr(vi), the dosage of nZVI@Fe_3_O_4_ was 3 g L^−1^, Cr(vi) was removed by more than 60% after 1 h and about 80% after 36 h (Fig. 6(b)). Lowering the ratio of nZVI@Fe_3_O_4_ to Cr(vi) strengthens competition for limited surface reactive sites, leading to site saturation and subsequent reduction in removal efficiency.^40^

To analyze the adsorption process of Cr(vi) on the surface of nZVI@Fe_3_O_4_, two kinetic models, namely the pseudo-first-order (PFO) and pseudo-second-order (PSO) equations, were used (Fig. 6(b) and (e)). The mathematical expressions of the PFO and PSO equations are described in the ESI,† and the Table S1† presents the regression correlation coefficients (*R*^2^) computed for the fitted curves. The PSO kinetic model demonstrated better fitting to describe the adsorption of Cr(vi) onto nZVI@Fe_3_O_4_ than the PFO, as evidenced by the higher *R*^2^ values.^24,41^ These results suggest that chemisorption, which is driven by valence forces or electron transfer, rather than boundary layer resistance, is the primary mechanism governing Cr(vi) adsorption onto the nZVI@Fe_3_O_4_ surface.^42,43^ The PSO kinetic model are summarized in Table 1. The first five sets of fitted data reveal that as the concentration increases, the adsorption speed of Cr(vi) by nZVI@Fe_3_O_4_ decreases, as reflected by the decrease in *k*_2_ values. Passivation commonly occurs between high concentration of Cr(vi) and Fe-based materials, where the formation of Cr precipitates can wrap around the active site and reduce the accessibility and/or affinity for Cr(vi) adsorption.^44^ However, even though the adsorption rate decreases with increasing initial concentration, the *q*_e_ increases in the process, suggesting that the wrapping on the active site does not affect the final uptake of Cr(vi) by nZVI@Fe_3_O_4_. The catalytic surface sites provided by Fe_3_O_4_, allowing for a sustained interaction between nZVI@Fe_3_O_4_ and Cr(vi), which reduces the passivation effect.^18^

**Table 1:** PSO kinetic parameters for Cr(vi) removal by nZVI@Fe_3_O_4_

Initial Cr(vi) concentration (mg L^−1^)	nZVI@Fe_3_O_4_ dosage (g L^−1^)	*q*_e_ (mg g^−1^)	*k*_2_ (g mg^−1^ min^−1^)
20	1.0	20.19	0.0120495
40		39.02	0.0017382
60		45.85	0.0008573
80		50.51	0.0006695
200	1.0	54.42	0.0002711
	2.0	48.35	0.0002949
	3.0	47.62	0.0003467

Moreover, it also can be found in Fig. 6(b) and (e) that the real data points show a step change between 120 min and 720 min which cannot be well captured by the proposed adsorption model, especially with high initial concentrations and low doses. It can be hypothesized that the adsorption of Cr(vi) at high concentrations involves not only electrostatic interactions between nZVI@Fe_3_O_4_ and Cr(vi), but also intra-particle diffusion processes.^2^

To explore the mechanisms of adsorption–diffusion, the intra-particle diffusion model was employed to analyze the kinetic data. Based on the results applied to this model, multistage adsorption of the removal process was noted, suggesting that this process may consist of two steps before reaching equilibrium, as indicated by Fig. 6(c) and (f). Obviously to see that the rate of adsorption is more rapid during the first step because Cr(vi) diffuses through the boundary layer and adsorbs on the nZVI@Fe_3_O_4_ surface. While the adsorption rate decreases significantly after 120 min, which is the second step, and intra-particle diffusion may be the main rate-controlling step. Additionally, it should be noted that the fitted curves for both stages did not originate from the origin of the coordinate system, suggesting that the adsorption process is not solely governed by intraparticle diffusion but may also involve some surface complexation reactions. This multistage adsorption indicates that intraparticle diffusion alone cannot be the sole rate-controlling step, and suggests the involvement of multiple mechanisms in the adsorption process.

#### 3.3.3. Adsorption thermodynamics

**Fig. 7:**
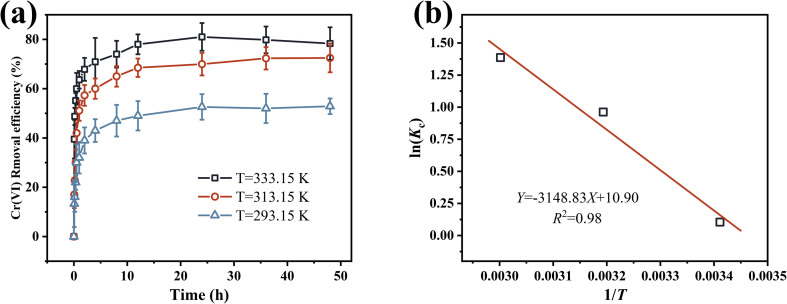
(a) Effect of temperature on Cr(vi) adsorption by nZVI@Fe_3_O_4_; (b) plot of *K*_C_*vs.* 1/*T* for determination of reaction enthalpy and entropy change of Cr(vi) removal by nZVI@Fe_3_O_4_.

Cr(vi) adsorption by nZVI@Fe_3_O_4_ was studied at different temperatures (293.15 K, 313.15 K, 333.15 K), Fig. 7 displays the results that correspond to the aforementioned analysis. The removal efficiency of Cr(vi) by nZVI@Fe_3_O_4_ increases significantly from 52.86% to 78.31% when the temperature is raised from 293.15 K to 333.15 K (Fig. 7(a)). The increase in reaction temperature results in higher removal efficiency, suggesting an exothermic adsorption process.^45^ On the one hand, the warming of the solution (<333.15 K) intensifies the diffusion of oxygen, leading to accelerated corrosion of nZVI@Fe_3_O_4_ and the creation of a larger surface area, which allows for more contact between active sites and Cr(vi), resulting in an increase in removal efficiency. In another light, an increase in temperature facilitates the movement of Cr(vi) from the solution to the nZVI@Fe_3_O_4_, as more molecules become activated and diffusion is enhanced, which increases the accessibility of active sites on the surface of nZVI@Fe_3_O_4_.^46^ From the linear relationship between ln(*k*_C_) and 1/*T* demonstrated in the Van't Hoff plot of Fig. 7(b), the correlation fitting results and calculated Gibbs free energy change (Δ*G*) are presented in Table 2, with the relevant formulae documented in ESI.†

**Table 2:** Thermodynamic parameters for the adsorption of Cr(vi) on nZVI@Fe_3_O_4_

*T* (K)	Δ*S* (J mol^−1^ K^−1^)	Δ*H* (kJ mol^−1^)	Δ*G* (kJ mol^−1^)
293.15	90.62	26.18	−0.25
313.15			−2.50
333.15			−3.84

The calculated Δ*G* values are consistently negative, indicating the feasibility of the removal process. With increasing temperature, Δ*G* decreases, suggesting an increase in the spontaneity of the reaction. The positive determine enthalpy change (Δ*H*) obtained from fitting the data suggest that the removal process is a heat-absorbing chemisorption process, which is consistent with the experimental observations that the removal efficiency increases with temperature. The positive entropy change (Δ*S*) values suggest that there is a structural change between the adsorbate and the adsorbent, reflecting the strong affinity of nZVI@Fe_3_O_4_ for Cr(vi). Such positive Δ*S* values are often attributed to the dehydration of metal ions and rupture of the hydration shell.^47^

#### 3.3.4. Adsorption isotherms

**Fig. 8:**
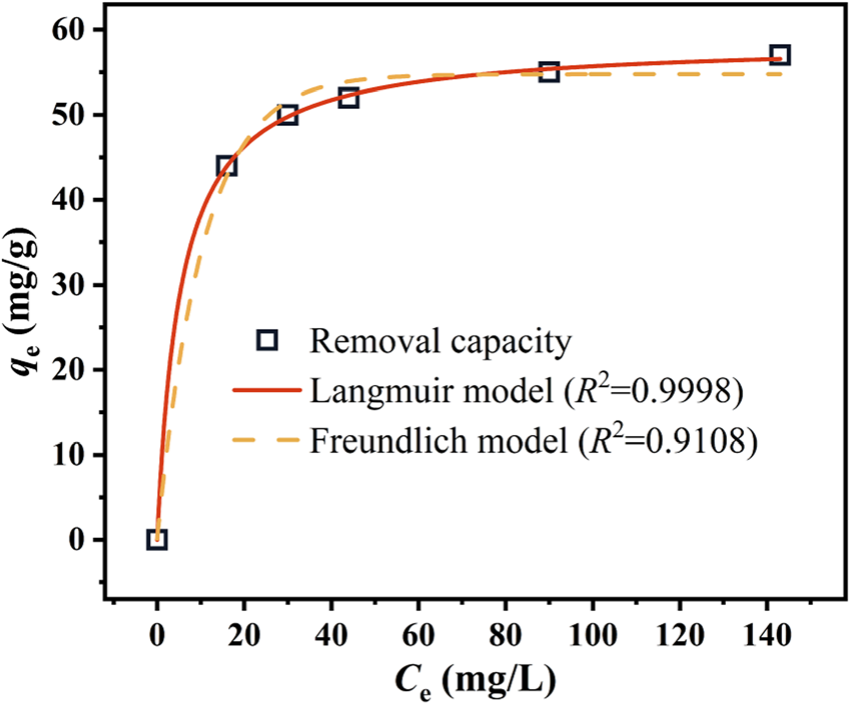
Equilibrium model of Cr(vi) removal by nZVI@Fe_3_O_4_.

Adsorption isotherms can be analyzed using two commonly used models. One model assumes a homogeneous surface with a limited number of identical sites and is referred to as the Langmuir model. The other model is more empirical and allows for multilayer adsorption, and is called the Freundlich model. There equations and other pertinent formulae are documented in the ESI.† In this study, the data were obtained from the results of equilibrium in kinetic studies, from which adsorption isotherms at 25 °C were obtained according to Langmuir and Freundlich model, an illustration of this relationship is presented in Fig. 8.

The higher *R*^2^ value of Langmuir model in Fig. 8(a) demonstrates that the removal of Cr(vi) by nZVI@Fe_3_O_4_ is mainly monolayer adsorption, and the calculated maximum adsorption capacity (*q*_max_) is 58.67 mg L^−1^. Comparison was made between nZVI@Fe_3_O_4_ and other Fe-based adsorbents reported in the literature (Table 3), the as-synthesized nZVI@Fe_3_O_4_ has a high adsorption capacity. Considering its cheap raw materials and simple preparation conditions, nZVI@Fe_3_O_4_ nanocomposites proved its potential practical value in Cr(vi) removal.

**Table 3:** Comparison of Cr(vi) removal capacity of Fe-based adsorbents

Number	Adsorbent	Equilibrium time	pH	*q*_max_	References
1	nZVI@Fe_3_O_4_	24 h	6.0	58.67	This work
2	nZVI–Fe_3_O_4_ nanocomposites	<2 h	3.0	100.00	*J. Colloid Interface Sci.*, 2012, **369**, 460–469 (ref. 18)
3		<2 h	8.0	29.43	
4	nZVI–graphene/Fe_3_O_4_	—	8.0	66.20	*J. Colloid Interface Sci.*, 2014, **417**, 51–59 (ref. 43)
5	Fe_3_O_4_ micron-spheres	60 h	3.0	43.48	*Chem.–Eur. J.*, 2012, **18**, 13418–13426 (ref. 48)
6	AVT–nZVI	—	5.0	59.17	*Chemosphere*, 2019, **218**, 458–467 (ref. 49)
7	nZVI–BC	—	4.0	58.82	*Chemosphere*, 2018, **207**, 50–59 (ref. 50)
8	BC–Fe	24 h	2.8	16.30	*J. Environ. Manage.*, 2022, **316**, 115260 (ref. 51)
9	nZVI–PCNFs	6 h	7.0	13.20	*RSC Adv.*, 2022, **12**, 8178–8187 (ref. 52)

The Langmuir isotherm has a key parameter known as *R*_L_, which characterizes the adsorption process of the adsorbent. The computed RL value of 0.016 indicates an advantageous adsorption process.

### 3.4. Removal of real electroplating wastewater

**Fig. 9:**
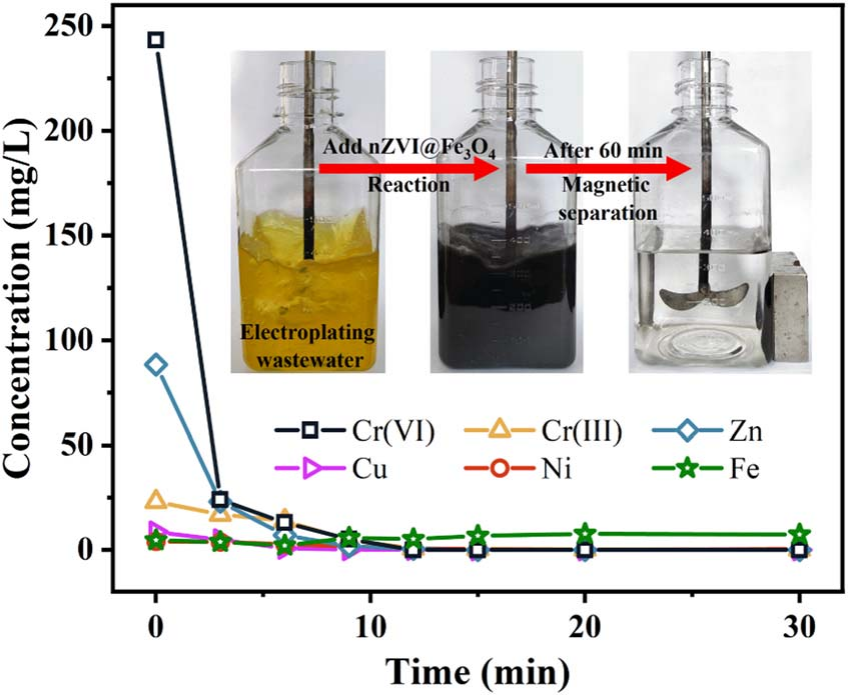
Removal of real electroplating wastewater using nZVI@Fe_3_O_4_.

This study explored the effectiveness of nZVI@Fe_3_O_4_ in removing Cr(vi) and other heavy metal ions from authentic electroplating wastewater. As showed in Fig. 9 and Table S2,† 1.5 g nZVI@Fe_3_O_4_ was added to 300 mL real plating wastewater for treatment, and the reaction was stirred in air to complete the removal process within 30 min. The magnetic properties of nZVI@Fe_3_O_4_ allowed for easy separation from the treated electroplating wastewater using a magnet, and it is found that 100% of Cr(vi) and 98.76% of Cr(iii) are removed, and the removal of other heavy metals is above 89%. Although the concentration of total Fe increased, it still remained within the desired range. The experimental findings indicate that the application of nZVI@Fe_3_O_4_ is highly effective in eliminating heavy metal ions from actual electroplating wastewater.

### 3.5. Reaction mechanism

**Fig. 10:**
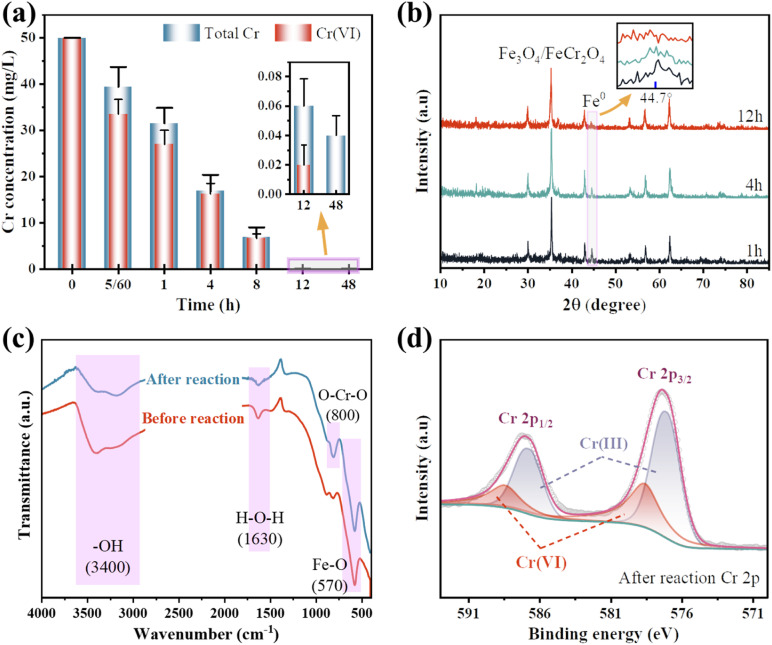
(a) The concentrations of total Cr and Cr(vi) in solution during the removal process; (b) the XRD patterns of nZVI@Fe_3_O_4_ during the adsorption; (c) FTIR spectra before and after the reaction of nZVI@Fe_3_O_4_ with Cr(vi); (d) Cr 2p XPS pattern after the reaction.

Fig. 10(a) and (b) illustrate the variations in total Cr and Cr(vi) concentrations in solution and the XRD patterns of nZVI@Fe_3_O_4_ during adsorption. It can be observed that the total concentration of both total Cr and Cr(vi) decreases with increasing adsorption time. Moreover, the α-Fe diffraction peak keeps weakening (the amplified portion in Fig. 10(b)). This indicates that the reduction of Cr(vi) occurs mainly on the nZVI@Fe_3_O_4_ rather than in the solution, and the nZVI has been consumed in the Cr(vi) removal process.

Fig. 10(c) displays the absorption peak at 570 cm^−1^ is believed to be related to the stretching vibration of the tetrahedral group (Fe–O) of nZVI@Fe_3_O_4_, while the peaks at 3400 and 1630 cm^−1^ are caused by the surface –OH.^23,53^ After the reaction, the peaks at about 800 cm^−1^ is observed which can be attributed to the antisymmetric Cr–O–Cr stretching, indicating the formation of a Cr-nZVI@Fe_3_O_4_ complex.^54^ This suggests that Cr(vi) has been efficiently captured onto the nZVI@Fe_3_O_4_ composites. Furthermore, a detailed XPS investigation of the nZVI@Fe_3_O_4_ composites after reaction in the Cr 2p region was performed, as given in Fig. 10(d). The peaks at 588.5 eV for Cr2p 1/2 and 578.6 eV for Cr 2p 3/2 are characteristic of Cr(vi) species, respectively, while the peaks at 586.7 and 577.2 eV belong to the corresponding peaks of Cr(iii) species.^55^ The results indicate that the reaction system involves both the adsorption and reduction of Cr(vi). The reaction can be expressed by eqn (6)–(9):

6 3Fe^0^ + 2HCrO_4_^−^ + 14H^+^ → 3Fe^2+^ + 2Cr^3+^ + 8H_2_O

7 3Fe^0^ + 2CrO_4_^2−^ + 16H^+^ → 3Fe^2+^ + 2Cr^3+^ + 8H_2_O

8 3Fe^2+^ + HCrO_4_^−^ + 7H^+^ → Cr^3+^ + 3Fe^3+^ + 4H_2_O

9 3Fe^2+^ + 2CrO_4_^2−^ + 8H^+^ → 3Fe^3+^ + Cr^3+^ + 4H_2_O

**Fig. 11:**
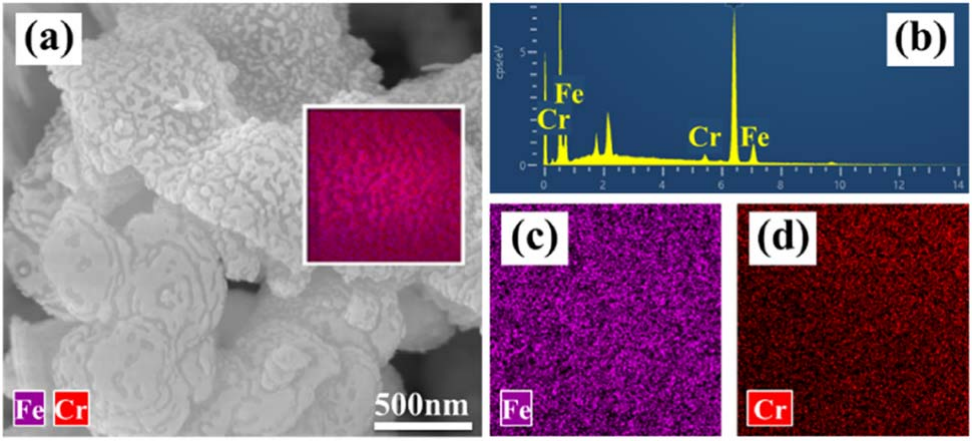
Characterization of nZVI@Fe_3_O_4_ after reaction with Cr(vi): (a) FESEM-EDS images, (b) EDS point scan, and EDS mapping of (c) Fe and (d) Cr elemental distribution.

The micrographs and elemental distribution of nZVI@Fe_3_O_4_ composite after reaction were characterized. It is evident that the surface roughness of nZVI@Fe_3_O_4_ increased and speckled corrosion products were generated after reaction with Cr(vi) (Fig. 11(a)). The EDS point scan displays that the surface of nZVI@Fe_3_O_4_ is coated with Fe, O and Cr (Fig. 11(b)), with Fe and Cr being distributed in the same position (Fig. 11(c) and (d)). Previous researches have indicated that the reaction between Cr(vi) and nZVI as well as Fe_3_O_4_ produces Fe/Cr oxygen hydroxyl precipitates that stick to the surface (eqn (10)).^44,48^

10 *x*Cr^3+^ + (1 − *x*)Fe^3+^ + 3H_2_O → Cr_*x*_Fe_(1−*x*)_(OH)_3_ + 3H^+^

The literature suggests that the combination of nZVI with Fe_3_O_4_ has shown improved results compared to using nZVI alone. This is because the Fe_3_O_4_ has an octahedral spinel structure, which can serve as a good redox site. On one hand, the surface of Fe_3_O_4_ contains Fe(ii), which acts as an active reduction site to reduce and detoxify contaminants. During this reaction process, Fe(ii) oxidizes itself to Fe(iii).^19,56^ On the other hand, Fe_3_O_4_ has a narrow band gap of 0.1 eV and metallic properties, which facilitates the transfer of electrons released by nZVI to the surface of Fe_3_O_4_.^18^ This promotes the regeneration of Fe(iii) to Fe(ii) and provides an additional channel for the transfer of electrons in addition to direct contact between the contaminant and nZVI.^18^ Therefore, the combination of nZVI and Fe_3_O_4_ ensures continuous reactions between the material and the contaminant, which leads to better results compared to using nZVI alone (eqn (11)).^20,57^

11 2Fe^3+^ + Fe^0^ → 3Fe^2+^

**Fig. 12:**
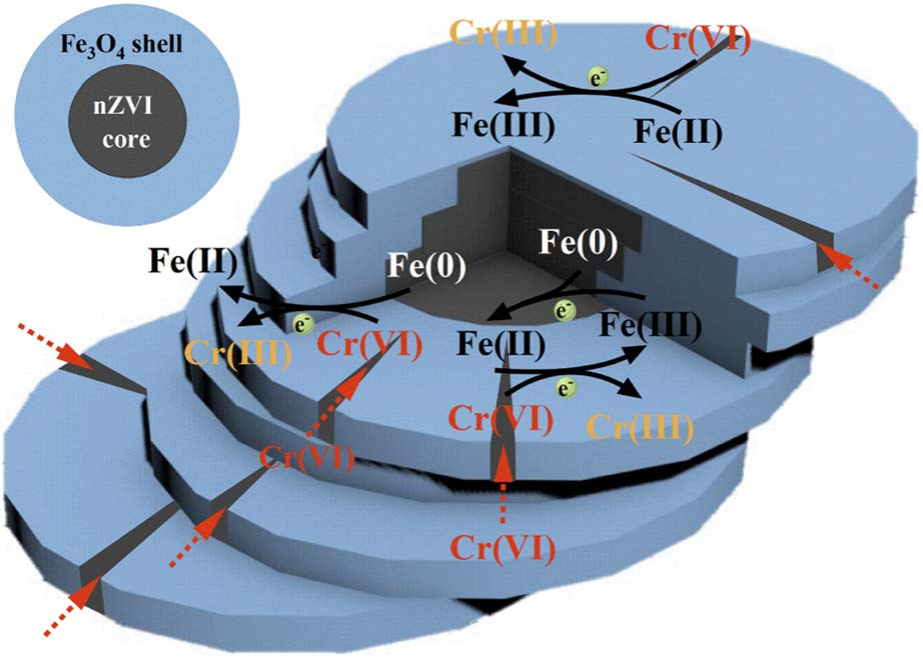
Schematic of Cr(vi) removal mechanism.

Based on the above discussion and analysis, it can be concluded that the main reaction process of nZVI@Fe_3_O_4_ in capturing Cr(vi) in solution can be described in Fig. 12. The as-synthesized nZVI@Fe_3_O_4_ has a large surface area, which allows for rapid capture of Cr(vi). Then part of the captured Cr(vi) attaches to Fe_3_O_4_ and is reduced and/or immobilized by Fe(ii). The cleavage structure of nZVI@Fe_3_O_4_ provides more active sites and opportunities for Cr(vi) to intrude inward. After that, Part of the intruded Cr(vi) has the opportunity to be directly reduced to Cr(iii) by nZVI. In addition, the nZVI in the core can reduce the oxidized Fe(iii) on the surface of Fe_3_O_4_ to Fe(ii), providing the additional channel for electron transfer. This ensures the long-term reactivity and ideal adsorption capacity of nZVI@Fe_3_O_4_ for Cr(vi) removal. Overall, the reaction process of nZVI@Fe_3_O_4_ with Cr(vi) is a complex process involving multiple steps of adsorption and reduction, which is facilitated by the unique properties of the material.

## 4. Conclusions

This study aimed to prepare core@shell structured nZVI@Fe_3_O_4_ nanocomposite for its potential application in removing Cr(vi) from aqueous solutions. The method involves a simple and low-cost process of Fe(ii) disproportionation reaction using NaOH at 150 °C for 0.5 h, which produced larger particles composed of nanosheets of Fe_3_O_4_ wrapped around nZVI. The resulting material exhibits excellent magnetic separation properties, as well as large surface area of fissure structures. The kinetic and thermodynamic studies indicate that the adsorption process follows the pseudo-second-order and Langmuir models, respectively, suggesting a spontaneous, heat-absorbing, and chemisorption process. The *q*_max_ of nZVI@Fe_3_O_4_ for Cr(vi) was calculated by the model to be 58.67 mg g^−1^ at pH = 6.0.

Moreover, the main mechanism of Cr(vi) removal by nZVI@Fe_3_O_4_ is attributed to adsorption, reduction, and co-precipitation reactions. The cleavage structure provides active sites for Cr(vi) immobilization and reduction, and the reduction of Cr(vi) to Cr(iii) is also facilitated by the electrons in nZVI through Fe_3_O_4_, which may be the main pathway for electron transfer and Cr(vi) detoxification. In conclusion this study demonstrate that nZVI@Fe_3_O_4_ prepared by Fe(ii) disproportionation reaction is a promising, cost-effective material for environmental and industrial wastewater treatment due to its excellent magnetic recovery properties and efficient removal of Cr(vi) and other heavy metal ions.

## Author contributions

Chuan He: conceptualization, methodology, material preparation, data analysis, writing – original draft; Yarong Ding: investigation, visualization; Canhua Li: supervision & project administration; Wang Yan: data analysis; Aiqin Mao: writing – review & editing; Shuxian Wei: investigation; Minghui Li: supervision.

## Conflicts of interest

There are no conflicts to declare.

## Supplementary Material

RA-013-D3RA03133K-s001
