# Supplementary material for: Cost-effective core@shell structured zero-valent iron nanoparticles @ magnetic (nZVI@Fe3O4) for Cr(vi) removal from aqueous solutions: preparation by disproportionation of Fe(ii)
Source: RSC Adv. 2023 Sep 8;13(38):26983–94. doi: 10.1039/d3ra03133k (PMC10485737; doi:10.1039/d3ra03133k)
Supplement: RA-013-D3RA03133K-s001 [file RA-013-D3RA03133K-s001.pdf]

Electronic Supplementary Material (ESI) for RSC Advances.
This journal is © The Royal Society of Chemistry 2023

Cost-effective Core@Shell structured zero-valent iron nanoparticles @ magnetic (nZVI@Fe_3O_4) for Cr(VI) removal from aqueous solutions: preparation by disproportionation of Fe(II)

Chuan He,[1] Yarong Ding,[1] Canhua Li,[1,3*], Aiqin Mao,[2] Shuxian Wei[1] and Minghui Li[1]

1. College of Metallurgical Engineering, Anhui University of Technology, Ma'anshan 243000, China.

2. School of Materials Science and Engineering, Anhui University of Technology, Ma'anshan, 243000, China.

3. Xuancheng Industrial Technology Research Institute, Anhui University of Technology, Xuancheng, 242002, China.

* Corresponding authors: Canhua Li E-mail: licanhua1979@163.com

Supporting Information

Analytical methods

Field emission scanning electron microscopy (FESEM, MIRA3, TESCAN) was utilized for characterizing the surface morphology of as-synthesized nZVI@Fe_3O_4， while high resolution transmission electron microscopy (HRTEM, Talos F200X, FEI) were employed for analysing the fine microstructure. nZVI@Fe_3O_4 and different reaction times with Cr(VI) were immediately subjected to X-ray diffraction (XRD, D8 ADVANCE, BRUKER) analysis. The combined elemental state of nZVI@Fe_3O_4 before and after the adsorption of Cr(VI) was analysed by X-ray photoelectron spectroscopy (XPS, ESCALAB Xi^+, ThermoFischer). The samples were analysed in depth by ion sputtering with an Ar^+ ion sputter gun. N_2 adsorption-desorption isotherms were recorded utilizing a surface area porosity analyser (JW-TB440, JWGB), using the Brunauer-Emmett-Teller (BET) method, the specific surface area was calculated at adsorption data points in the relative pressure P/P_0 range of 0.10 to 0.30. The magnetic characteristic of the as-synthesized nZVI@Fe_3O_4 was tested by vibrating sample magnetometer (VSM, 7404, Lake Shore) at room temperature. The possible functional groups of the material were tested using Fourier transform infrared spectroscopy (FTIR, Nicolet iS20, ThermoFischer). Testing the zeta potential of the materials at different pH involved using the solid addition method with a zeta potential analyser (Zetasizer Nano ZS90, Malvern). Using a spectrophotometer (721N, Shanghai INESA Analytical Instrument) measured the concentration of Cr(VI) at a wavelength of 540 nm through the 1,5-diphenylcarbazide method. For the analysis of total chromium and other metals in aqueous solutions, inductively coupled plasma emission spectrometry (ICPS-7510 PLUS, SHIMADZU) was employed. Calculating the difference between the total Cr and Cr(VI) concentrations provided us with the concentration of Cr(III).

Characterization of nZVI@Fe_3O_4

The average crystalline size of particles can be calculated by Scherrer's formula, as shown in Eq. (1)

$$D = \frac{\mathrm{K}\lambda}{\beta \cos\theta} \tag{1}$$

where D is the crystallite domain size (nm); K is the constant (0.89); λ is the diffracted ray wavelength (1.54 Å, nm); β is the full width at half maximum (FWHM) of the diffraction peak; θ is the Bragg's angle.

Adsorption kinetics models

The pseudo-first-order (PFO) was expressed:

$$q_t = q_e(1 - e^{-k_1 t}) \tag{1}$$

where q_e (mg g^{-1}) represents the amount of Cr(VI) adsorbed at equilibrium and k_1 (min^{-1}) is the PFO rate constant.

The linear form of the PSO can be expressed:

$$q_t = \frac{k_2 q_e^2 t}{1 + k_2 q_e t} \tag{2}$$

where k_2 (mg g^{-1} min^{-1}) is the PSO rate constant.

The intra-particle diffusion model:

$$q_t = k_d \sqrt{t} + I \tag{3}$$

Here, k_d and I represent the internal diffusion model constants and the adsorption constants related to the boundary layer thickness, respectively.

Thermodynamic model

A Van't Hoff plot of kC vs. 1/T was constructed according to the following equation:

$$\ln k_C = \ln \frac{C_{ac}}{C_e} = \frac{\Delta S}{R} - \frac{\Delta H}{RT} \tag{4}$$

here, k_C represents the equilibrium constant, C_{ac} represents the equilibrium concentration of Cr(VI) on the nZVI@Fe_3O_4 (mg L^{-1}), R is the ideal gas constant (8.314 J mol^{-1} K^{-1}), and T is the absolute temperature (K).

In Figure 7(b), the Van't Hoff plot shows a linear relationship and the slope and intercept of the fitted results can be used to determine enthalpy change (ΔH) and entropy change (ΔS), respectively. The Gibbs free energy change (ΔG) was calculated:

$$\Delta G = -RT \ln k_C \tag{5}$$

Adsorption isotherm models

The Langmuir and Freundlich adsorption isotherm models were used to describe the Cr(VI) adsorption isotherm data, and the two isotherm equations could be expressed as Eq. 6 and Eq. 7:

$$q_e = \frac{q_{max} k_L C_e}{1 + k_L C_e} \tag{6}$$

$$q_e = k_F C_e^{1/n} \tag{7}$$

Where q_{max} (mg g^{-1}) denotes the maximum adsorptive performance of nZVI@Fe_3O_4, and the Langmuir adsorption constant (k_L) and Freundlich affinity coefficient (k_F) are parameters that characterize of an adsorbent, while $1/n$ is a factor that describes the heterogeneity of the adsorption process.

An important feature of the Langmuir isotherm is the R_L, a factor that explains the nature of the adsorption process of the selected adsorbent, which can be calculated using Eq. 8. The constant R_L >1 indicates that the adsorption process is unfavorable; If R_L =1, the process is linear, and for 0> R_L <1, the adsorption process is favorable; Whereas R_L =0, the process is not reversible.

$$R_L = \frac{1}{1 + k_L C_e} \tag{8}$$

Table S1 Regression correlation coefficients for pseudo-first-order and pseudo-second-order models

Initial Cr(VI) concentration (mg L^{-1})	nZVI@Fe_3O_4 dosage (g L^{-1})	R^2	
		Pseudo-first-order kinetic model	Pseudo-second-order kinetic model
20	1.0	0.9634	0.9972
40		0.8516	0.9404
60		0.8516	0.8911
80		0.7061	0.9180
200	1.0	0.9339	0.9781
	2.0	0.8145	0.9162
	3.0	0.8097	0.9059

Table S2. Metal ion concentration in real electroplating wastewater before and after nZVI@Fe_3O_4 treatment.

Ions	Before reaction (mg L^{-1})	After reaction mg (mg L^{-1})	Removal efficiency (%)
Cr(VI)	243.23	0	100
Cr(III)	23.11343	0.2875	98.76
Zn	88.49	0	100
Cu	8.64286	0.07142	99.17
Ni	4.12434	0.4375	89.39
Fe	4.64286	7.42857	-
pH	3.9	5.8	-
